# Effect of two-doses of 3-nitrooxypropanol (3-NOP) on methane emissions, performance, rumen microbiome, and metabolomics in Nellore cattle

**DOI:** 10.1093/jas/skag068

**Published:** 2026-02-27

**Authors:** Bruna R Amancio, Elaine Magnani, Alanne T Nunes, Thiago H Silva, Cristina Cortinhas, Victor V Carvalho, Luis F M Tamassia, Reto Zihlmann, Alexandre Berndt, Juliana O S Marcatto, Nara R B Consolo, Pedro D B Benedeti, Renata H Branco, Eduardo M Paula

**Affiliations:** Institute of Animal Science, Sustainable Livestock Center, Sao Jose do Rio Preto, SP 15025-970, Brazil; Present address: Center for Nuclear Energy in Agriculture, University of São Paulo, Piracicaba, SP 13416-000, Brazil; Institute of Animal Science, Sustainable Livestock Center, Sao Jose do Rio Preto, SP 15025-970, Brazil; Department of Veterinary Medicine, School of Animal Science and Food Engineering (FZEA), University of São Paulo, Pirassununga, SP 13635-900, Brazil; Institute of Animal Science, Sustainable Livestock Center, Sao Jose do Rio Preto, SP 15025-970, Brazil; dsm-firmenich Nutritional Products, Department of Innovation & Applied Science, SP 15025-970, Brazil; dsm-firmenich Nutritional Products, Department of Innovation & Applied Science, SP 15025-970, Brazil; dsm-firmenich, Department of Animal Nutrition and Health, Kaiseraugst 4303, Switzerland; dsm-firmenich, Department of Animal Nutrition and Health, Kaiseraugst 4303, Switzerland; Research and Development, EMBRAPA Southeast Livestock, Sao Carlos, SP 13560-970, Brazil; Department of Global Climate Change and Agricultural, Embrapa Environment, Jaguariuna, SP 13820-000, Brazil; Department of Nutrition and Animal Production, School of Veterinary Medicine and Animal Science, University of São Paulo, Pirassununga, SP 13635-900, Brazil; Department of Animal Sciences, Santa Catarina State University, Chapecó, SC 89815-630, Brazil; Institute of Animal Science, Sustainable Livestock Center, Sao Jose do Rio Preto, SP 15025-970, Brazil; Institute of Animal Science, Sustainable Livestock Center, Sao Jose do Rio Preto, SP 15025-970, Brazil; Present address: AgNext, Department of Animal Sciences, Colorado State University, Fort Collins, CO 80523, United States

**Keywords:** Bovaer, 3-NOP, feed additives, greenhouse gases, sustainability

## Abstract

This study evaluated the effects of two doses of 3-nitrooxypropanol (**3-NOP**) on methane (**CH_4_**) emissions, performance, dry matter (**DM**) intake, apparent digestibility, rumen microbiome and metabolomic profile of Nellore cattle fed a high-concentrate finishing finishing diet. Seventy-five 20-month-old Nellore bulls, 361.6 ± 30.08 kg body weight (**BW**), were individually housed with ad libitum access to feed and water. Animals were distributed in a completely randomized study design, with three treatments and 25 animals per treatment, which were: 1) **CON**, control (basal diet + mineral premix without 3-NOP), 2) **3-NOP65** (Basal diet + mineral premix + 65 mg 3-NOP/kg of DM), 3) **3-NOP85** (Basal diet + mineral premix + 85 mg 3-NOP/kg of DM). The 115-d trial included a 3-wk adaptation period with increasing dietary concentrate levels from 50% to 88%. Enteric CH_4_ emissions were measured using the sulfur hexafluoride (SF_6_) tracer gas technique. Supplementation with 3-NOP had no detrimental effect on final BW (*P *= 0.89) and average daily gain (ADG; *P *= 0.94), but DM intake increased linearly with 3-NOP inclusion (*P *= 0.05). Methane emissions (g/d) were reduced by 13.2% and 26.7% in the 3-NOP65 and 3-NOP85 groups, respectively (*P* < 0.05), without adverse effects on animal health. Rumen microbiome analysis revealed a quadratic response in the relative abundance of the phylum *Euryarchaeota* (*P *= 0.01). Metabolomic analysis indicated significant changes in amino acid and energy metabolism, with proline, arginine, and threonine identified as key discriminant metabolites (VIP > 1) in the 3-NOP85 group. These findings demonstrate that 3-NOP supplementation effectively reduces CH_4_ emissions in a dose-dependent manner while maintaining animal performance and health.

## Graphical Abstract

**Graphical Abstract:**
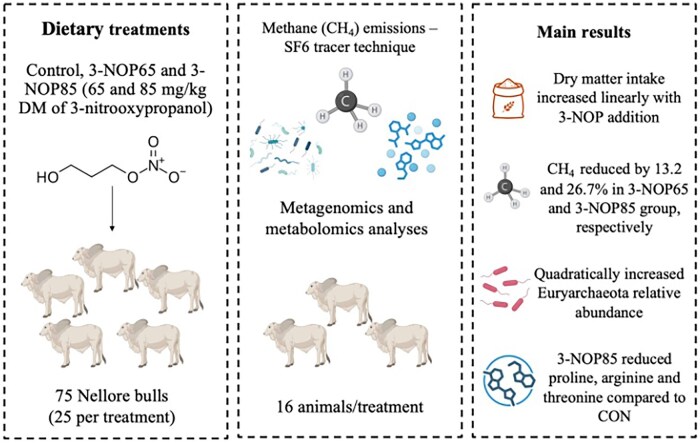

## Teaser

Supplementing Nellore cattle fed a high concentrate finishing diet with 65 mg/kg and 85 mg/kg of DM of 3-nitrooxypropanol (3-NOP) significantly reduces methane emissions, enhancing the sustainability of beef production. This strategy altered fermentation dynamics and nutrient metabolism without compromising animal health and performance.

## Introduction

Improving livestock productivity while reducing environmental impact is essential for achieving ecological sustainability and ensuring long-term economic resilience. In this context, mitigating enteric methane (**CH_4_**) emissions is regarded as one of the most cost‑effective strategies to slow global warming (Beauchemin et al. 2022). Consequently, reducing CH_4_ output from ruminants has become a central focus of global climate‑change mitigation efforts. Among the available mitigation strategies, the use of feed additives that inhibit methanogenesis has shown potential for reducing enteric CH_4_ emissions in the short-to-medium term (Króliczewska et al. 2023). Within this category, 3-nitrooxypropanol (**3-NOP**) has emerged as one of the most effective and consistent feed additives for reducing CH_4_ emissions in both beef and dairy cattle (Jayanegara et al. 2018; Alemu et al. 2021; Yu et al. 2021; Gruninger et al. 2022; Pitta et al. 2022; Araújo et al. 2023). Different studies have reported average CH_4_ reductions of approximately 39% in dairy cattle and 22.2% in beef cattle with 3-NOP supplementation (Dijkstra et al. 2018). However, most studies on 3-NOP have been conducted in North America, Asia, and Europe, with limited data available from South America. Araújo et al. (2023) reported CH_4_ reductions of up to 52.7% in Nellore bulls fed 100 mg/kg dry matter (DM) of 3-NOP in a typical finishing diet. Similarly, a study in Australia also observed reductions of up to 90% in Angus steers supplemented with 50 to 125 mg/kg DM of 3-NOP, without adverse effects on rumen fermentation or animal performance (Almeida et al. 2023).

Despite these promising results, there is a lack of studies evaluating the effects of 3-NOP at inclusion levels below 100 mg/kg DM in Brazilian beef production systems, particularly in Nellore cattle. Lower inclusion rates may offer a more economically viable option for producers, especially in developing regions. Thus, we hypothesized that 3-NOP supplementation at levels below 100 mg/kg DM would effectively reduce CH_4_ emissions without compromising animal performance. Therefore, the objective of this study was to evaluate the effects of two dose rates of 3-NOP (65 or 85 mg/kg of DM) on CH_4_ emissions, performance, apparent total tract digestibility, rumen microbiome, and metabolomic profile of Nellore cattle fed a typical Brazilian finishing diet.

## Materials and methods

### Experimental location and ethical approval

The experiment was conducted at the Animal Science Research Institute of São Paulo, Beef Cattle Researcher Center, located in Sertãozinho, SP, Brazil. All procedures involving animals were approved by the Animal Use Ethics Committee of the institute (Protocol No. 362-2022) and followed the guidelines of the Institutional Animal Care and Use Committee, in accordance with Brazilian legislation on the ethical use of animals in research (State law 11,977, Sao Paulo).

### Animals, experimental design, and feeding

The experiment lasted 115 d (December 1st, 2022 to March 26th, 2023), including 21-d adaptation period. Seventy-five 20-month-old Nellore bulls, 361.6 ± 30.08 body weight (**BW**) ± SD were sourced from a commercial farm and housed individually in concrete pens with ad libitum access to feed and water. Upon arrival, bulls were weighed and received standard health treatments, including ivermectin (1 mL/50 kg of BW; Ivomec Gold, Boehringer Ingelheim), vitamin A, D, and E (5 mL/animal; Calbos Animal health, Brazil), a clostridial vaccine (3 mL/animal), and a follow-up albendazole dose (1 mL/33 kg of BW) after 30 d. A completely randomized design was used with three treatments (n = 25 per treatment): 1) **CON**, control (basal diet + mineral premix without 3-NOP), 2) **3-NOP65** (Basal diet + mineral premix + 65 mg 3-NOP/kg of DM; Bovaer^®^ - dsm-firmenich Nutritional Products), 3) **3-NOP85** (Basal diet + mineral premix DM+ 85 mg 3-NOP/kg of DM; Bovaer^®^ - dsm-firmenich Nutritional Products). Monensin was added in all dietary treatments at 25 mg/kg of DM.

The adaptation period included three sequential diets over 21 d, with increasing concentrate levels: wk 1 (from 50% to 60% concentrate, from d0 to d7), wk 2 (from 60% to 75% concentrate, from d7 to d14), and wk 3 (from 75% to 88% concentrate, from d14 to d21), all on a DM basis. The 3-NOP supplementations began at the beginning of wk 2, thus, DM intake data from wk 1 were excluded from analysis. The finishing diet consisted of 11.8% sugarcane bagasse and 88.2% concentrate (DM basis; Table 1). Diets were formulated to support an average daily gain (ADG) of 1.5 kg/d, according to NASEM (2016) guidelines.

**Table 1:** Ingredients and chemical composition of experimental diets containing different levels of 3-nitrooxypropanol (3-NOP) during the finishing phase.

Item	3-NOP, mg/kg DM		
	0	65	85
**Ingredient, % (dry matter basis)**			
** Sugarcane bagasse**	12	12	12
** Ground corn**	36	36	36
** Citrus pulp**	21	21	21
** Cottonseed meal**	20	20	20
** Dried distiller grain**	6	6	6
** Premix^1^**	5	5	5
**Chemical composition, % of DM**			
** Dry matter, % of as fed**	68.4	69.8	69.6
** Organic matter**	90.5	90.5	90.5
** Crude protein**	16.3	16.3	16.4
** Ether extract**	4.04	4.04	4.05
** Neutral detergent fiber**	33.8	33.8	33.9
** Metabolizable energy, Mcal/kg^2^**	2.95	2.95	2.95
** Net energy for gain, Mcal/kg^2^**	1.32	1.32	1.32
** 3-NOP, mg/kg of Ground corn + Premix**	–	174	232

1 Provided (per kg of DM): 242.5 g of calcium; 18 g of phosphorus; 70 g of sodium; 17 g of magnesium; 23 g of sulfur; 14 mg of chromium; 1700.0 mg of zinc; 455 mg of copper; 1210.0 mg of manganese; 38 mg of iodine; 20 mg of cobalt; 14 mg of selenium; 83400.0 IU of vitamin A; 16680.0 IU of vitamin D; 170.0 IU of vitamin E, 410 mg/kg of monensin.

2 Estimated using NASEM (2016) guidelines.

The 3-NOP was first mixed with ground corn and the mineral premix using a premix feed mill, then incorporated into the total mixed ration (TMR) using a TMR mixer (Vertmix; Casale, Sao Carlos, SP, Brazil). Diets were offered *ad libitum* once daily, and intake was recorded by weighing feed offered and refusals each day. Feed amounts were adjusted to maintain at least 3% orts throughout the experiment.

### Performance evaluation

Animals were weighed after a 16-h fasting period on d 0 and again on day 115 (end of the experimental period). Additional intermediate weighing was conducted without fasting on Days 21 (end of adaptation), 53, and 84. The ADG was calculated over the entire 115-d period. Efficiency of feed utilization was calculated as the ratio of ADG to DMI. Carcass gain was calculated using the following formula, assuming an initial carcass dressing of 50%:


$$\text{Carcass}\;\text{gain}\;(\text{kg})=\frac{\left(\text{FBW},\;\text{kg}\times \text{Carcass}\;\text{dressing},\;\%)-(\text{IBW},\;\text{kg}\times 0.5\right)}{\text{days}\;\text{on}\;\text{feed}}$$

where, FBW = final BW, IBW = initial BW.

### Enteric CH_4_ emissions

CH_4_ emissions were measured using the sulfur hexafluoride (SF_6_) tracer gas technique, as described by (Johnson and Johnson 1995). Sixteen animals per treatment were randomly selected for gas sampling; these same animals were also used to evaluate apparent digestibility, rumen microbiome composition, and metabolomic profiles. To minimize stress and ensure accurate measurements, animals remained in their individual pens during the entire sampling process. Each animal was fitted with a gas collection halter 30 d prior to the sampling period to allow for adaptation of the animal to wearing the halter. Sulfur hexafluoride permeation capsules were inserted 10 d before sampling began. Gas collection was conducted over five consecutive days (Days 104 to 110 of the experimental period), with continuous 24-h sampling, totaling 120 h per animal (Berndt et al. 2014).

Expired and eructated gas samples were collected in evacuated stainless-steel canisters attached to the halters. Canisters were replaced every 24 h, before feeding, resulting in five gas samples per animal. Additionally, ambient air samples were collected daily using three canisters per day to correct for background CH_4_ and SF_6_ concentrations.

At the end of the sampling period, all gas samples were sent to Embrapa Environment (Jaguariúna, SP, Brazil) for analysis. Gas concentrations were determined using a gas chromatograph (HP6890, Agilent Technologies, San Jose, CA, USA) equipped with a flame ionization detector (FID) set at 280 °C and a Plot HP-Al/M megabore column (0.53 μm, 30 m) for CH_4_ detection. An electron capture detector was used for SF_6_ analysis.

CH_4_ emissions rates (QCH_4_) were calculated based on the ratio of CH_4_ to SF_6_ concentrations in the collected samples, adjusted for the known SF_6_ release rate from the permeation tubes and corrected for ambient gas concentrations. Emissions were expressed in multiple forms to assess environmental and production efficiency: grams CH_4_/day, grams CH_4_/kg DMI, grams CH_4_/kg ADG, grams CH_4_/kg carcass gain, grams CH_4_/kg hot carcass weight (HCW). The DMI intake data from the CH_4_ sampling period were used for all CH_4_ calculations.

### Apparent digestibility and chemical analysis

Feed ingredients, orts, and feces were sampled weekly for apparent digestibility estimations. Fecal samples were collected over three consecutive days during three distinct periods of the experimental phase at 6 h before and 6 h after feeding: Days 32 to 34, 64 to 66, and 96 to 98. Samples were collected directly from the rectum or immediately after defecation to avoid contamination. All fecal samples were dried in a forced-air oven at 60 °C for 72 h and ground to pass through a 1-mm screen using a Wiley mill. Equal DM portions from each daily subsample were pooled per animal to create one composite sample per period. These composites were used to determine chemical composition and estimate apparent total tract nutrient digestibility.

All samples were analyzed for DM (method 930.15; AOAC, 1990), ash (method 942.05; AOAC, 1990), and crude protein (Dumatherm^®^; GerhardtGmbH & Co, Konigswinter, Germany; method 990.13; AOAC, 2005). The organic matter (OM) was calculated as the difference between DM and ash contents. For neutral detergent fiber (NDF), samples were treated with alpha thermo-stable amylase without sodium sulfite (Van Soest et al. 1991), using a method adapted for a Fiber Analyzer (TE 149; Tecnal^®^, Piracicaba, SP, Brazil). Fecal samples were also analyzed for indigestible ADF, which is the ADF residue remaining after a 12-d in situ incubation, which was used as an internal marker to estimate fecal output and apparent total-tract digestibility (Cochran et al. 1986; Huhtanen et al. 1994). Gross energy (GE) determinations were performed using an adiabatic bomb calorimeter (Ika^®^, model C200, Germany).

### Rumen microbiome

#### Collection, DNA extraction, targeted library preparation

At slaughter, approximately 100 g of ruminal content (a mixture of liquid and solid phases) were collected from the dorsal, central, and ventral regions of the rumen. Samples were immediately placed into a thermal container maintained at 4 °C and transported to a commercial laboratory for DNA extraction and sequencing. Samples were processed and analyzed using the ZymoBIOMICS Targeted Sequencing Service (Zymo Research, Irvine, CA). The DNA was extracted using the ZymoBIOMICS-96 MagBead DNA Kit on an automated platform. In some cases, the ZymoBIOMICS DNA Miniprep Kit or DNA Microprep Kit was used, particularly for low-biomass samples, to ensure higher DNA concentration through reduced elution volumes.

Bacterial 16S ribosomal RNA gene sequencing was performed using the Quick-16S™ NGS Library Prep Kit (Zymo Research, Irvine, CA). The bacterial 16S primers amplified the V3-V4 region of the 16S rRNA gene. These primers have been custom designed to provide the best coverage of the 16S gene while maintaining high sensitivity. Fungal ITS gene-targeted sequencing was performed using the Quick-16S™ NGS Library Prep Kit with custom ITS2 primers substituted for 16S primers. The sequencing library was prepared using an innovative library preparation process in which PCR reactions were performed in real-time PCR machines to control cycles and therefore limit PCR chimera formation. The final PCR products were quantified with qPCR fluorescence readings and pooled together based on equal molarity. The final pooled library was cleaned with the Select-a-Size DNA Clean & Concentrator™ (Zymo Research, Irvine, CA), then quantified with TapeStation^®^ (Agilent Technologies, Santa Clara, CA) and Qubit^®^ (Thermo Fisher Scientific, Waltham, WA).

#### Sequencing and bioinformatic analysis

The final library was sequenced on Illumina MiSeq with a v3 reagent kit (600 cycles). The sequencing was performed with 10% PhiX spike-in. Unique amplicon sequences variants were inferred from raw reads using the DADA2 pipeline (Callahan et al. 2016). Potential sequencing errors and chimeric sequences were also removed with the Dada2 pipeline. Taxonomy assignment was performed using Uclust from Qiime v.1.9.1 with the Zymo Research Database, a 16S database that is internally designed and curated, as reference. Composition visualization, alpha-diversity, and beta-diversity analyses were performed with Qiime v.1.9.1 (Caporaso et al. 2010). Other analyses such as heatmaps, Taxa2ASV Deomposer, and PCoA plots were performed with internal scripts.

#### Absolute abundance quantification

A quantitative real-time PCR was set up with a standard curve. The standard curve was made with plasmid DNA containing one copy of the 16S gene and one copy of the fungal ITS2 region prepared in 10-fold serial dilutions. The primers used were the same as those used in Targeted Library Preparation. The equation generated by the plasmid DNA standard curve was used to calculate the number of gene copies in the reaction for each sample. The PCR input volume was used to calculate the number of gene copies per microliter in each DNA sample. The resulting values are shown in the gene copies column of the absolute abundance results table.

The number of genome copies per microliter DNA sample (genome copies) was calculated by dividing the gene copy number by an assumed number of gene copies per genome. The value used for 16S copies per genome is 4. The value used for ITS copies per genome is 200. The amount of DNA per microliter DNA sample (DNA_ng) was calculated using an assumed genome size of 4.64 x 106 bp, the genome size of Escherichia coli, for 16S samples, or an assumed genome size of 1.20 x 107 bp, the genome size of Saccharomyces cerevisiae, for ITS samples. This calculation is shown below:


Calculated Total DNA = (Calculated Total Genome Copies×Assumed Genome Size (4.64×106 bp)×Average Molecular Weight of a DNA bp×(660 g/mole/bp))/(Avogadros Number (6.022 x 1023/mole))"

### Animal health

The incidence and severity of rumenitis were evaluated by a trained technician using the methodology proposed by Bigham and McManus (1975). Rumen lesions were scored on a scale from 0 to 10 points, where each point represents 10% of the rumen surface area affected. Liver health was assessed based on the presence and severity of abscesses, following the classification system proposed by Brown et al. (1975). Liver scores were assigned as follows: 0 (No abscesses present), A− (One or two small abscesses, <2.5 cm in diameter, or healed abscess scars), A (Two to four active abscesses, each slightly smaller than 2.5 cm in diameter), and A+ (One or more large abscesses, >2.5 cm in diameter, often with adhesions to the diaphragm).

### Metabolomic analyses

#### Metabolite extraction and NMR sample preparation

Blood samples were collected on d 115 of the experimental period, concurrently with BW measurements. Ten milliliters of blood were drawn from the jugular vein into plain vacuum tubes (BD Vacutainer, São Paulo, SP, Brazil) and placed on ice until centrifugation, which were immediately preceded at 3,000 × g for 15 min at 4 °C to separate the serum, which was aliquoted into labeled 2 mL microtubes and stored at −80 °C until analysis.

Serum samples were thawed at room temperature for 30 min prior to metabolite extraction. A double-phase extraction protocol was performed as described by Da Costa et al. (2020). Briefly, 500 µL of serum was transferred to a 2 mL microcentrifuge tube and mixed with 480 µL of ice-cold acetone:methanol (1:1, v/v), followed by being vortexed. Then, 400 µL of ice-cold chloroform was added, and the mixture was vortexed again. After incubating on ice for 10 min to precipitate proteins, samples were centrifuged at 10,000 × g for 10 min at 4 °C. The resulting supernatant was reconstituted in 500 µL of phosphate buffer (0.1 M, pH 7.4) prepared in D_2_O containing 0.5 mM DSS‑d_6_ (4,4-dimethyl-4-silapentane-1-sulfonic acid) as an internal chemical-shift and quantitation standard. Samples were vortexed, centrifuged at 14,000 × g for 5 min, and transferred into 5 mm NMR tubes.

#### NMR data acquisition and processing

Proton nuclear magnetic resonance (^1H NMR) spectra were acquired using a Bruker AVANCE III HD 600 MHz spectrometer (14.1 T) equipped with a 5 mm z-gradient probe at 298 K, following the protocol described by Cônsolo et al. (2022). The noesygppr1d pulse sequence was used for water suppression.

For each sample, 128 scans were collected over a spectral width ∼20 ppm (0.22 Hz/point), with an acquisition time of 4.50 s, a relaxation delay of 25 s, and a 90-pulse width of 13.1 µs. Spectral processing was performed using TopSpin 3.7 (Bruker BioSpin). Chemical shifts were referenced to the DSS methyl resonance at 0.00 ppm. A 0.3 Hz exponential line broadening was applied prior to Fourier transformation. Automatic phase and baseline corrections were applied, with manual adjustments as needed. Metabolite identification and quantification were performed using the Profiler module of Chenomx NMR Suite Professional v10 (Chenomx Inc., Edmonton, Canada). A total of 38 metabolites were assigned by matching spectral peaks to the Chenomx 1D spectral library. Peak integrals were quantified relative to the DSS internal standard (0.5 mM).

### Statistical analysis

#### Performance, digestibility, CH_4_ emissions, and rumen microbiome

All statistical analyses were performed using SAS software (version 9.4; SAS Institute Inc., Cary, NC, USA). Data were analyzed as a completely randomized design using analysis of variance. Treatment means were compared using the least significant difference test. Polynomial regression analyses were performed to evaluate linear and quadratic effects of 3-NOP inclusion for additive levels. Because the additive levels (0, 65, and 85 mg/kg) were non‑equidistant, orthogonal polynomial coefficients were generated in SAS using the IML procedure based on the actual numeric dose values. The resulting linear and quadratic contrast coefficients were then incorporated into the mixed model to test dose‑response patterns. Statistical significance was declared at *P *≤ 0.05, and trends were discussed when 0.05 < *P *< 0.10.

#### Liver abscess and rumen score

Ordinal health outcomes (rumenitis and liver abscess scores) were analyzed using a generalized linear mixed model (GLMM) with a multinomial logistic regression and a cumulative logit link function. This approach allowed estimation of the cumulative probability of achieving a given score across treatments. The ESTIMATE statement in SAS was used to compute the cumulative probability of lower severity scores under each treatment.

#### Metabolomic data

Metabolomic data were processed and analyzed using MetaboAnalyst 6.0. Raw concentration tables were square root-transformed and range-scaled. Missing values were imputed using a k‑nearest neighbors’ algorithm based on sample similarity. To identify treatment‑related metabolic shifts, a pairwise comparison was performed between the control group (CON) and the 3-NOP85 group and selected based on phenotypic divergence. Partial least squares discriminant analysis (PLS‑DA) was used to visualize group separation and detect potential outliers. Model quality was assessed using leave-one-out cross-validation, with R^2^ (goodness of fit) and Q^2^ (predictive ability) used as performance metrics. Metabolites with variable importance in projection (VIP) scores > 1 were considered key discriminators between treatments. Finally, enrichment analysis was conducted to identify biological processes associated with these metabolites, using a significant threshold of *P *< 0.10.

## Results

### Animal performance and dry matter intake

Final BW and ADG were not affected by 3-NOP supplementation (*P *= 0.89 and *P *= 0.94, respectively, Table 2). However, during both finishing (*P *= 0.05) and total (*P *= 0.003) periods (109d; 88:12 concentrate: roughage), DMI increased linearly with increasing 3-NOP levels (*P *= 0.05). Despite this increase in DMI, efficiency of feed utilization (gain: feed ratio) was not affected (*P *= 0.28). The HCW, carcass gain, and dressing percentage also remained unaffected by 3-NOP supplementation (*P *> 0.10).

**Table 2:** Least square means of performance parameters in Nellore bulls receiving different levels of 3-nitrooxypropanol (3-NOP).

Item	3-NOP, mg/kg DM			SEM	*P*-value		
	0	65	85		Trt	Lin	Quad
**n**	25	25	25				
**Initial body weight, kg**	360	362	363	6.09	0.93	0.70	0.99
**Final body weight, kg**	528	534	532	8.61	0.89	0.70	0.77
**Average daily gain, kg/d**	1.47	1.50	1.47	0.06	0.94	0.89	0.74
**Gain: Feed^2^**	0.136	0.132	0.127	0.004	0.24	0.13	0.47
**Dry matter intake, kg/d, finishing (24-109 d)**	10.7	11.4	11.8	0.382	0.14	0.05	0.79
**Dry matter intake, kg/d, total period (7-109 d)**	10.8^b^	11.5^a,b^	11.9^a^	0.267	0.01	0.003	0.70
**Hot carcass weight, kg**	302	302	304	5.10	0.93	0.85	0.73
**Carcass gain, kg/d**	0.92	0.85	0.89	0.027	0.21	0.21	0.20
**Dressing, %**	56.8	56.5	56.7	0.359	0.88	0.80	0.66

a–c Means within a row with different superscripts differ by LSD test (*P* ≤ 0.05).

### Apparent total tract digestibility

The CP digestibility was quadratically affected by 3-NOP inclusion (*P *= 0.04), with the highest digestibility observed at 85 mg/kg DM (Table 3). A quadratic trend was also observed for dry matter digestibility (*P *= 0.07), again peaking at the highest inclusion level of 3-NOP tested. No differences were observed for OM and NDF digestibility (*P *> 0.05).

**Table 3:** Least square means of total tract apparent digestibility in Nellore bulls receiving different levels of 3-nitrooxypropanol (3-NOP)

	3-NOP, mg/kg DM			SEM	*P*-value		
Item^1^	0	65	85		Trt	Lin	Quad
**Dry matter, %**	57.5	55.9	58.7	1.05	0.19	0.76	0.07
**Organic matter, %**	62.3	60.7	62.4	1.03	0.43	0.76	0.21
**Neutral detergent fiber, %**	32.7	33.2	34.9	1.09	0.28	0.17	0.42
**Crude protein, %**	64.5	61.8	65.1	1.17	0.12	0.81	0.04

### Methane emissions and ruminal microbiota

The DMI and gross energy intake during the CH_4_ collection period were not affected by 3-NOP supplementation (*P *> 0.10, Table 4). However, daily CH_4_ emissions decreased quadratically with increasing 3-NOP levels (*P *= 0.05). Emissions were reduced by 13.2% and 26.6% at 65 and 85 mg/kg DM of 3-NOP, respectively.

**Table 4:** Least square means of methane emissions in Nellore bulls receiving different levels of 3-nitrooxypropanol (3-NOP).

Item^1^		3-NOP, mg/kg DM			SEM	*P*-value		
		0	65	85		Trt	Lin	Quad
**n**		16	16	16	–	–	–	–
**Dry matter intake^2^, kg/d**		10.7	10.3	10.8	0.51	0.78	0.93	0.49
**Gross energy intake, Mcal/d**		47.7	47.1	51.5	2.34	0.36	0.40	0.25
**Methane emission**								
** CH_4_, g/d**		192^a^	166^b^	140^c^	5.14	<0.001	<0.01	0.05
** CH_4_, g/kg DMI**		17.2^a^	15.6^a^	13.3^b^	0.65	<0.001	<0.01	0.12
** GH_4_ energy loss, % GE intake**		5.24^a^	4.56^b^	3.90^c^	0.22	<0.001	<0.01	0.27
** CH_4_, g/kg ADG**		120^a^	105^b^	93.6^b^	4.62	<0.001	<0.01	0.37
** CH_4_, g/kg Carcass gain**		214^a^	198^a^	157^b^	11.1	0.002	0.003	0.06
** CH_4_, g/kg HCW**		0.638^a^	0.547^b^	0.462^c^	0.20	<0.001	<0.001	0.12

a–c Means within a row with different superscripts differ by LSD test (*P* ≤ 0.05).

d–f Means within a row with different superscripts differ by LSD test (0.05 < *P* < 0.10).

1 DMI = dry matter intake, GE = gross energy, ADG = average daily gain, HCW = hot carcass weight.

2 DMI and GE intake refer to the methane sampling period (5d).

CH_4_ yield (g CH_4_/kg DMI) decreased linearly (*P *< 0.001), with reductions of 9.2% and 22.3% for the of 3-NOP65 and of 3-NOP85 treatments, respectively. Similarly, the proportion of GE lost as CH_4_ decreased linearly (*P *< 0.001), with reductions of 12.9% and 25.6% for the two 3-NOP inclusion levels. CH_4_ intensity, expressed as CH_4_ per unit of ADG, carcass gain, and HCW, also decreased linearly (*P *< 0.001, *P *= 0.003, and *P *< 0.001, respectively). Notably, CH_4_ emissions per kg of HCW were reduced by 14.3% and 27.6% at 65 and 85 mg/kg DM, respectively.

No differences were observed in the absolute abundance of any phylum (*P *> 0.10), and the *Firmicutes* to *Bacteroidetes* ratio remained unchanged (*P *= 0.34, Table 5). However, the relative abundance of the *Euryarchaeota* was quadratically increased by 3-NOP supplementation (*P *= 0.01), with the highest value at 65 mg/kg DM. A quadratic trend was also observed for *Bacteroidetes* (*P *= 0.09), peaking at 85 mg/kg DM of 3-NOP.

**Table 5:** Absolute and relative abundance of ruminal microbial phyla in Nellore bulls receiving different levels of 3-nitrooxypropanol (3-NOP).

Item	3-NOP, mg/kg DM			SEM	*P*-value		
	0	65	85		Trt	Lin	Quad
**Absolute abundance, × 10^7^ ng/µL**							
** *Euryarchaeota***	3.14	3.72	3.62	0.71	0.82	0.57	0.81
** *Bacteroidetes***	37.4	38.0	45.9	6.67	0.62	0.46	0.50
** *Firmicutes***	45.0	56.4	59.2	9.42	0.54	0.27	0.95
** *Fibrobacteres***	0.93	1.14	1.04	0.29	0.74	0.72	0.50
** *Actinobacteria***	1.51	1.41	2.06	0.42	0.97	0.80	0.99
** *Proteobacteria***	2.33	2.04	2.31	0.48	0.87	0.97	0.61
** *Spirochaetae***	0.95	1.27	1.61	0.26	0.53	0.27	0.91
** Others**	9.44	8.18	10.4	1.67	0.65	0.89	0.36
***Firmicutes* to *Bacteroidetes* ratio**	1.46	1.49	1.31	0.12	0.34	0.42	0.23
**Relative abundance, %**							
** *Euryarchaeota***	2.69^a,b^	3.09^a^	2.46^b^	0.23	0.03	0.37	0.01
** *Bacteroidetes***	37.0	34.4	39.2	1.92	0.22	0.74	0.09
** *Firmicutes***	48.4	49.1	45.7	1.72	0.33	0.43	0.21
** *Fibrobacteres***	0.58	0.71	0.64	0.12	0.72	0.60	0.55
** *Actinobacteria***	1.54	1.14	1.89	0.42	0.97	0.80	0.99
** *Proteobacteria***	2.24	1.98	2.12	0.28	0.80	0.65	0.63
** *Spirochaetae***	1.10	1.17	1.35	0.13	0.41	0.24	0.50
** Others (<0.5%)**	8.13	8.03	7.49	0.51	0.66	0.46	0.55

a–c Means within a row with different superscripts differ by LSD test (*P* ≤ 0.05).

### Liver and rumen health

The 3-NOP supplementation had no significant effect on liver or rumen health (Table 6). The odds of lower liver abscess scores did not differ among treatments (*P *= 0.85), nor did the odds of lower rumenitis score (*P *= 0.18).

**Table 6:** Frequency and odds of liver abscess and rumenitis in Nellore bulls receiving different levels of 3-nitrooxypropanol (3-NOP).

Item	3-NOP, mg/kg DM			*P*-value
	0	65	85	
**Liver abscess score^1^, %**				
** 0**	37.5 _(0.34)_	25.0 _(0.27)_	25.0 _(0.27)_	0.54
** A−**	56.3 _(0.64)_	75.0 _(0.71)_	75.0 _(0.71)_	–
** A**	–	–	–	–
** A+**	6.25 _(0.02)_	0 _(0.02)_	0 _(0.02)_	–
** Odds ratio (95% CI)^2^**	Reference	1.43 _(0.31–6.65)_	1.43 _(0.31–6.65)_	0.86
**Rumenitis score^3^, %**				
** 0**	–	–	–	0.30
** 1**	43.8 _(0.42)_	12.5 _(0.18)_	25.0 _(0.23)_	–
** 2**	18.8 _(0.27)_	31.3 _(0.22)_	25.0 _(0.25)_	–
** 3**	12.5 _(0.09)_	12.5 _(0.11)_	6.3 _(0.11)_	–
** 4**	12.5 _(0.08)_	6.3 _(0.12)_	12.5 _(0.11)_	–
** 5**	6.3 _(0.04)_	12.5 _(0.08)_	0 _(0.07)_	–
** 6**	0 _(0.04)_	0 _(0.08)_	18.8 _(0.07)_	–
** 7**	6.3 _(0.03)_	12.5 _(0.08)_	0 _(0.07)_	–
** 8**	0 _(0.04)_	12.5 _(0.12)_	12.5 _(0.09)_	–
** 9**	–	–	–	–
** 10**	–	–	–	–
** Odds ratio (95% CI)^2^**	Reference	3.27 _(0.89-11.92)_	2.40 _(0.65-8.92)_	0.18

1 Frequency and probability of liver abscess scores: 0 (no abscesses), A− (1–2 small abscesses or scars), A (2–4 active abscesses), or A+ (large abscesses with adhesions).

2 Odds of having a low liver or rumenitis score in response to 3-NOP inclusion.

3 Rumenitis scores range from 0 (no lesions) to 10 (severe rumen wall damage).

### Metabolite identification

**Figure 1:**
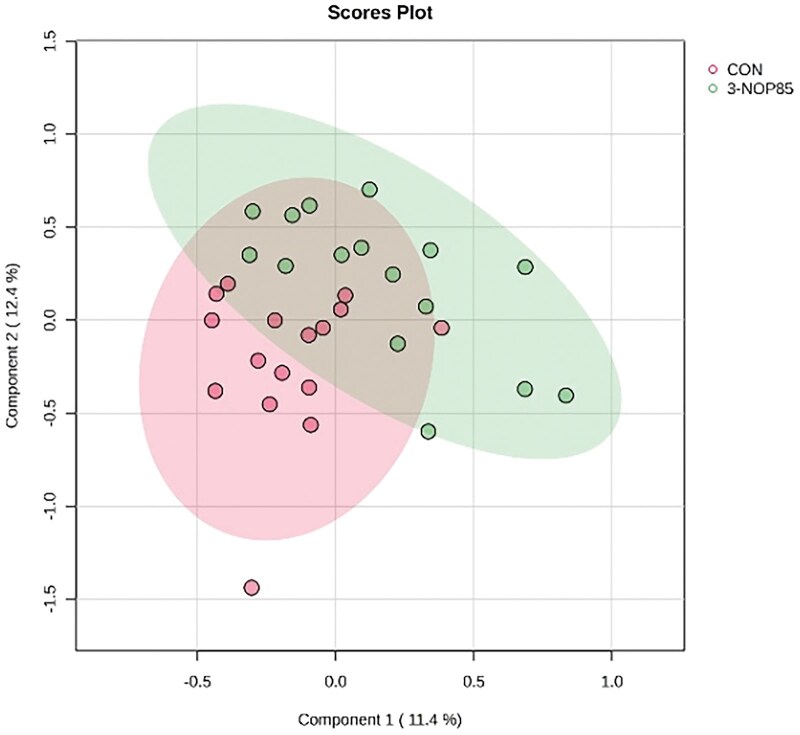
Partial least squares discriminant principal component analysis (PLS-DA) plot of serum metabolomic profiles in Nellore cattle from the control group (CON) and those supplemented with 3-nitrooxypropanol (3-NOP85). The plot highlights a clear separation between groups, indicating distinct metabolic signatures and highlighting the impact of 3-NOP supplementation on systemic metabolism.

**Figure 2:**
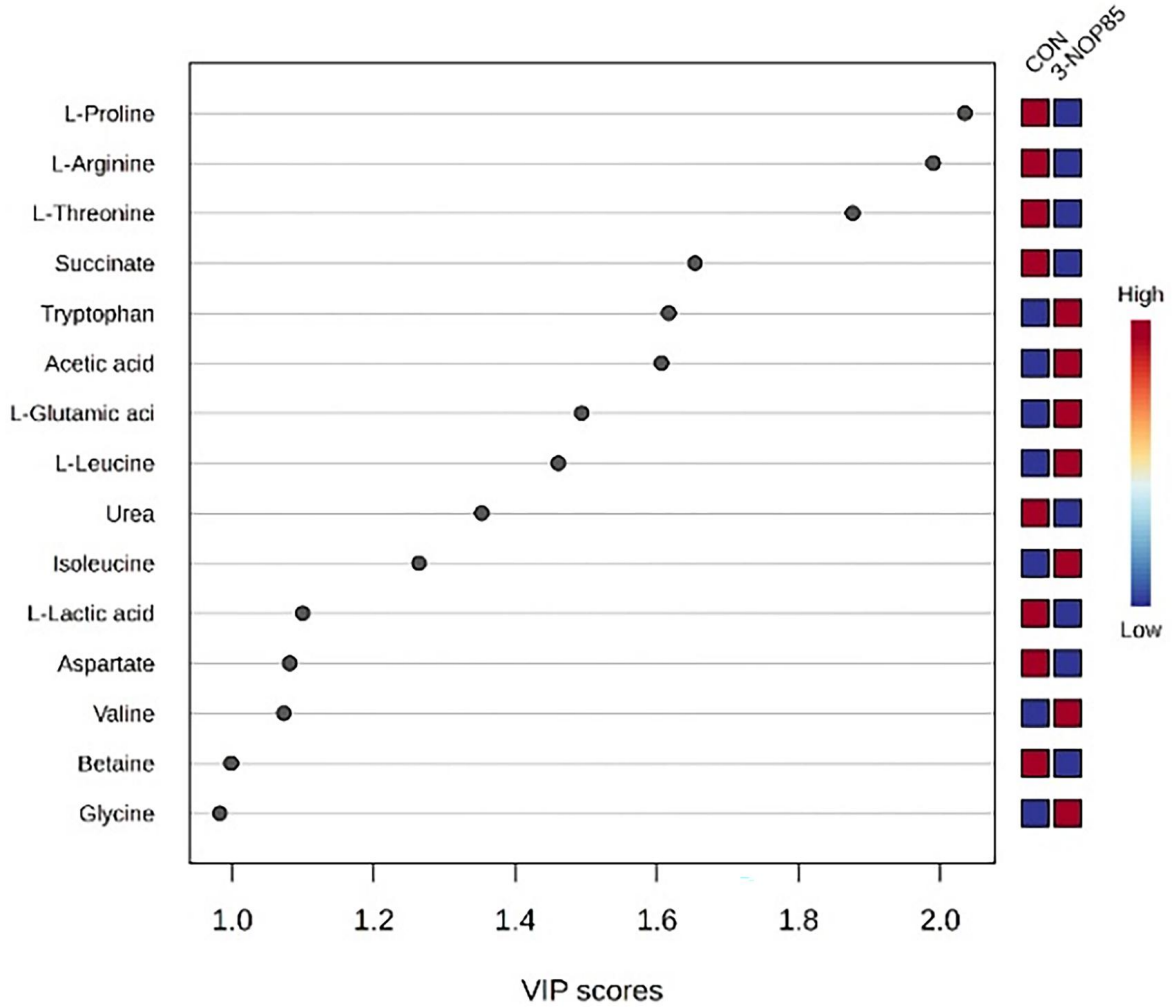
Variable Importance in Projection (VIP) scores for blood serum metabolites differentiating Nellore cattle from the control group (CON) and those supplemented with 3-nitrooxypropanol (3-NOP85). The VIP scores indicate the relative contribution of each metabolite group separation in the PLS-DA model, with higher scores reflecting greater discriminatory power. The color gradient (red to blue) represents the relative abundance of each metabolite, where red denotes higher concentrations in the 3-NOP85 group and blue indicates lower concentrations.

**Figure 3:**
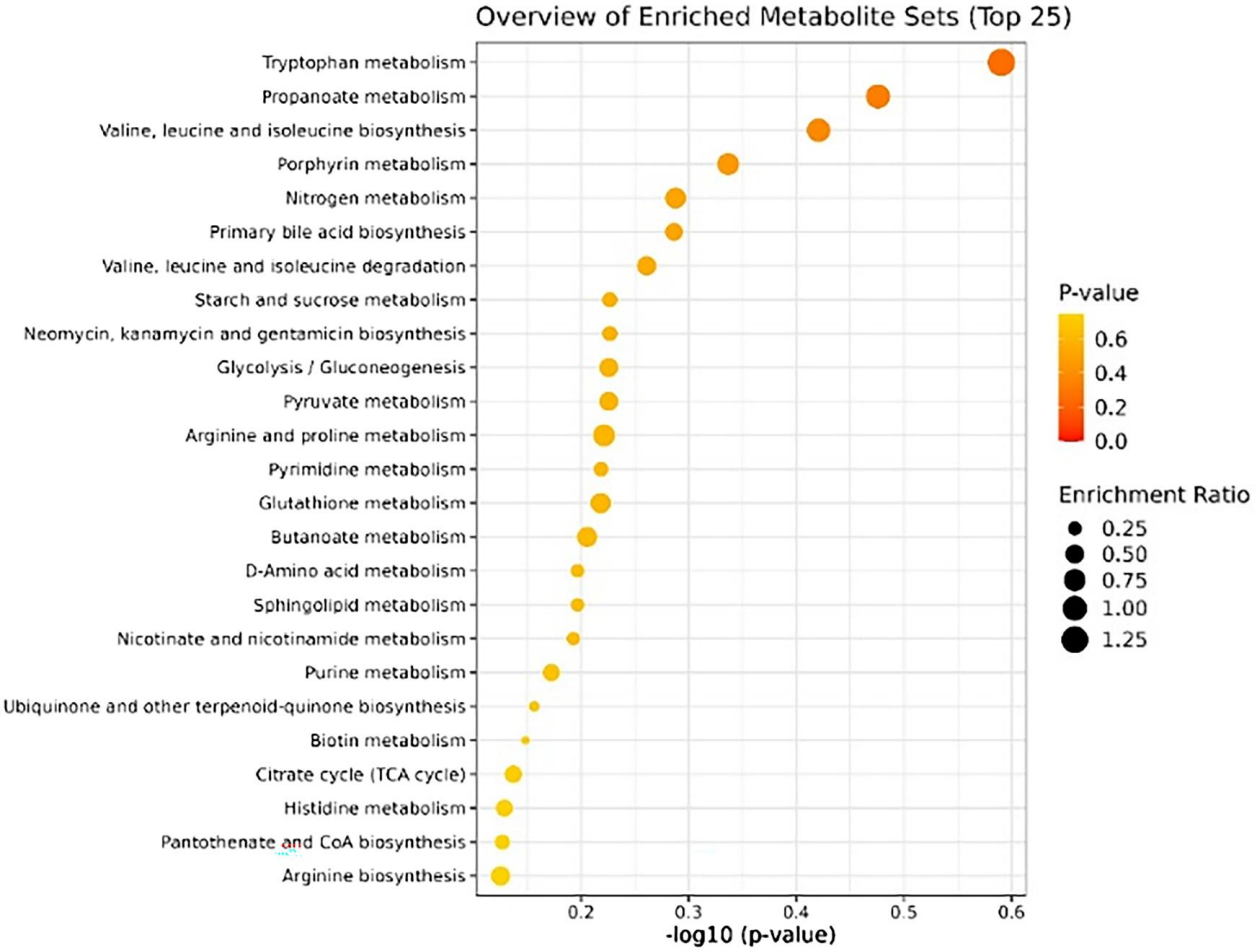
Enrichment analysis of serum metabolite pathways in Nellore cattle from the control group and those supplemented with 3-nitrooxypropanol (3-NOP85). The analysis highlights significantly altered metabolic pathways, including tryptophan metabolism, propanoate metabolism, and the biosynthesis of branched-chain amino acids (valine, leucine, and isoleucine), indicating shifts in amino acid and energy metabolism associated with 3-NOP supplementation.

The 3-NOP supplementation at 85 mg/kg DM significantly altered the serum metabolite profile compared to the control group. Partial least squares discriminant analysis (PLS-DA) revealed clear separation between the CON and 3-NOP85 groups (Figure 1), indicating distinct metabolic signatures. Key discriminant metabolites identified by VIP scores > 1 included L-proline, L-arginine, and L-threonine (Figure 2). Enrichment analysis of metabolite sets revealed significant alterations in several metabolic pathways, including tryptophan metabolism, propanoate metabolism, valine, leucine, and isoleucine biosynthesis (Figure 3). These results suggest that 3-NOP supplementation might modulate amino acid metabolism and energy-related pathways.

## Discussion

We hypothesized that dietary supplementation with 3-NOP at levels below 100 mg/kg DM would effectively reduce enteric CH_4_ emissions without compromising animal performance. The results confirmed this hypothesis: supplementation at 65 and 85 mg 3-NOP/kg DM significantly reduced CH_4_ emissions while maintaining animal health and productive performance.

The feed additive 3-NOP has been extensively studied for its potent inhibitory effect on methanogenesis in ruminants (Jayanegara et al. 2018; Alemu et al. 2021; Yu et al. 2021; Gruninger et al. 2022; Pitta et al. 2022; Araújo et al. 2023). This compound specifically targets methyl-coenzyme M reductase (MCR), a key enzyme in the final step of CH_4_ biosynthesis by methanogenic archaea. Mechanistically, 3-NOP binds to the active site of MCR and promotes the oxidation of the catalytic nickel ion from Ni^+^ to Ni^2+^, thereby temporarily inactivating the enzyme and halting the reduction of CO_2_ to CH_4_ via dissolved H_2_ (Duin et al. 2016). Although increasing doses of 3-NOP did not enhance growth performance in Nellore bulls fed a typical Brazilian finishing diet, the additive consistently reduced CH_4_ emissions. Additionally, there was a tendency for improved digestibility of DM and CP, suggesting potential shifts in ruminal fermentation dynamics.

These metabolic changes suggest that 3-NOP supplementation redirected systemic metabolism towards enhanced nutrient utilization, protein synthesis, and energy production, as revealed by distinct shifts in serum metabolite profiles. Notably, animals receiving 3-NOP85 exhibited a significant reduction in serum succinate levels compared to the control group. Succinate, a key intermediate in ruminal carbohydrate fermentation, plays a central role in microbial energy metabolism and serves as a precursor for propionate and pyruvate production (Nagaraja 2016). The observed decrease in succinate may indicate an accelerated flux through energy-generating pathways, contributing to improved metabolic efficiency.

In addition to energy metabolism, 3-NOP85 supplementation significantly influenced amino acid metabolism. Alterations in serum concentrations of proline, arginine, and threonine, amino acids essential for protein synthesis and energy metabolism, suggest enhanced nutrient utilization and metabolic regulation. Moreover, changes in branched-chain amino acids (BCAAs), including valine, leucine, and isoleucine, point to potential improvements in muscle protein synthesis and anabolic signaling pathways (Zhang et al. 2017; Imaz et al. 2022). These findings support the hypothesis that 3-NOP not only mitigates CH_4_ emissions but also promotes favorable shifts in host metabolism.

Even though no significant changes in animal performance or carcass weight were observed, the altered serum concentrations of proline and arginine, key intermediates in the urea cycle, may indicate a reduction in nitrogen excretion. Unfortunately, aspects of nitrogen metabolism were not measured in the scope of this study but should be considered in future studies. These changes are likely driven by changes in rumen fermentation and are crucial for supporting growth and maintaining overall animal health (Widmann et al. 2013; Shimizu et al. 2015; Gómez et al. 2022; Polizel et al. 2024). Additionally, the observed modulation of threonine levels, an amino acid involved in immune function and collagen synthesis (Defa et al. 1999; Li et al. 2007; Yu et al. 2020), may suggest that 3‑NOP85 supplementation has the potential to influence pathways associated with animal health. However, direct physiological effects cannot be inferred solely from serum metabolite data. Finally, despite indications that 3‑NOP supplementation affects metabolic pathways, it is important to note that no direct supporting measurements, such as blood urea nitrogen, ruminal VFA concentrations, ruminal pH, or digesta passage rate, were not evaluated in this study. These variables would provide critical mechanistic insights and should be included in future investigations to strengthen the interpretation of metabolic outcomes.

Our findings align with previous studies conducted under Brazilian production conditions, which similarly reported CH_4_ reductions without changes in animal performance (Alemu et al. 2021; Araújo et al. 2023). The present study was specifically designed to evaluate the efficacy of lower 3-NOP doses under Brazilian conditions, providing valuable insights into the potential for CH_4_ mitigation with minimal additive input.

In contrast to previous studies, we observed a novel, dose‐dependent linear increase in DMI with 3-NOP supplementation. This finding differs from earlier reports in which long-term 3-NOP supplementation has been associated with reduced DMI in beef cattle (Vyas et al. 2018; Kim et al. 2020). One proposed mechanism linking 3‑NOP to altered intake behavior involves increased ruminal H_2_ concentrations, which may shift fermentative pathways by downregulating H_2_‑producing routes and upregulating H_2_‑utilizing pathways, particularly those leading to propionate and acetate synthesis (Jayanegara et al. 2018; Melgar et al. 2020). This mechanism is consistent with the higher serum acetate concentrations observed in the 3-NOP85 group. Although carcass fat deposition was not measured in this study, increased acetate availability may facilitate the incorporation of specific fatty acids into adipose tissue, supporting findings by Pedrini et al. (2024) using the same commercial product.

The increased DMI observed in this study may also be related to changes in rumen fermentation and microbiome composition. Previous research has shown that 3-NOP can reduce total VFA production in the rumen (Kim et al. 2019a; Kim et al. 2020), potentially triggering a compensatory hyperphagic response. According to Allen (2000), reduced VFA concentrations in the reticulo-rumen and bloodstream may lead to earlier return of hunger and shorter inter-meal intervals, thereby increasing DMI. Consistent with this idea, we observed a linear increase in the relative abundance of the *Bacteroidetes* phylum with 3-NOP supplementation. These generalist microbes are capable of degrading a wide range of polysaccharides (Gharechahi et al. 2023) and are recognized as net H_2_ utilizers (Wolin et al. 1997; Gruninger et al. 2022). These microbial shifts may have contributed to the increased DMI observed in 3-NOP supplemented animals in this study. Another possible explanation, particularly relevant for cattle fed high‑concentrate diets, is that 3‑NOP may reduce the risk or severity of subacute ruminal acidosis (Kim et al. 2019a; Alemu et al. 2023; Orzuna-Orzuna et al. 2024), thereby supporting more stable intake patterns and potentially allowing higher peak DMI at times when low ruminal pH would typically restrict intake. Collectively, these findings suggest that higher degradability rates and increased rumen fluid pH may have occurred in the supplemented groups, although these parameters were not directly measured in this study.

Furthermore, nutrient digestion was affected, as evidenced by the increase in total‑tract digestibility of DM and CP, with digestibility values plateauing, or reaching their highest levels, in the group receiving 85 mg/kg. This pattern paralleled the trend observed for the *Bacteroidetes* phylum. These changes may have influenced the serum metabolomic profiles discussed earlier. Similarly, Romero-Perez et al. (2014) reported a trend toward a quadratic increase in DM digestibility in heifers supplemented with increasing levels of 3-NOP in a Latin square design. Likewise, Hristov et al. (2015) observed a quadratic increase in the digestibility of DM, OM, CP, and ADF when intermediate doses of 3-NOP (40 and 60 mg of 3-NOP/kg DM) were fed to dairy cattle. These shifts in rumen fermentation patterns may enhance feed energy utilization and improve nutrient digestibility, particularly in high-forage diets.

The findings of this study support the hypothesis that 3-NOP acts as an effective inhibitor of methanogenesis, as evidenced by the reduction in enteric CH_4_ emissions in Nellore bulls. Daily CH_4_ emissions were reduced by 13.2% and 26.7% with 3-NOP supplementation at 65 and 85 mg/kg DM, respectively. When expressed relative to DMI, CH_4_ emissions declined by 9.2% and 22.2% for the same doses, reflecting the increase in DMI. These reductions are consistent with previous reports, such as Dijkstra et al. (2018), who predicted a 22% decrease in CH_4_ emissions in beef cattle fed 123 mg/kg of DM of 3-NOP from a meta-analysis of data derived from peer-reviewed beef studies conducted with 3-NOP. Similarly, Hristov et al. (2015) reported CH_4_ reductions of 20, 25, and 29% in dairy cows supplemented with 40, 60, and 80 mg/kg DM of 3-NOP, respectively, using the SF_6_ tracer technique.

Recent study performed by Araújo et al. (2023) under Brazilian conditions also demonstrated significant CH_4_ mitigation in Nellore bulls. The reduction was by 52.7% and 36% during days 27 to 33 and 90 to 96 of the finishing period, respectively, when animals were supplemented with an average of 100 mg/kg DM of 3-NOP. On average, CH_4_ production declined by 43.9% across both sampling periods, a greater reduction than observed in the present study. This discrepancy may be attributed to the timing of CH_4_ measurements, which in our study were conducted only at the end of the finishing period, whereas Araújo et al. (2023) sampled at both the beginning and end. Romero-Perez et al. (2015) also reported variable CH_4_ reductions over a 112-d trial with beef heifers. Interestingly, CH_4_ emissions were higher during d 57 to 85 compared to earlier periods but declined again during d 85 to 112 to a level similar to the initial phase, suggesting no long-term microbial adaptation to 3-NOP. Although such temporal dynamics are intriguing, it is important to acknowledge that CH_4_ was measured at only a single time point in the present study, limiting our ability to evaluate potential adaptation effects. Nevertheless, in the present study, GE losses as CH_4_ were reduced by 12.9% and 25.6% with 3-NOP supplementation at 65 and 85 mg/kg DM, respectively, indicating improved dietary efficiency. However, this reduction in energy loss as CH_4_ did not translate into improved animal performance.

Methanogens, which belong to the phylum *Euryarchaeota* within the domain Archaea, are responsible for CH_4_ production in anaerobic environments (Hedderich and Whitman 2013). In this study, the relative abundance of *Euryarchaeota* was quadratically affected by 3-NOP supplementation. Although functional activity of these microorganisms was not evaluated, this shift in abundance may help explain the observed reductions in CH_4_ emissions by suggesting a potential suppression of methanogenesis and a decline in methanogen populations. This interpretation is consistent with previous in vitro and in vivo studies showing that 3-NOP selectively inhibits methanogens with minimal impact on other rumen microbes (Duin et al. 2016; Gruninger et al. 2022). Additionally, the relative abundance of the phylum *Bacteroidetes* increased with 3-NOP supplementation at 85 mg/kg DM, although the *Firmicutes*:*Bacteroidetes* ratio remained unchanged. *Bacteroidetes* are known net H_2_ utilizers (Wolin et al. 1997), and the elevated H_2_ levels associated with 3-NOP supplementation (Gruninger et al. 2022) may have created favorable conditions for their proliferation (Gharechahi et al. 2023). These microbial shifts further support the observed changes in rumen fermentation and CH_4_ mitigation. Nevertheless, it is important to emphasize that rumen fluid samples for microbiota and metabolite analyses were collected after a 16‑h fasting period. Fasting reduces the availability of fermentable substrates in the rumen, which in turn decreases VFA production, increases ruminal pH, and may reduce overall microbial biomass (Kim et al. 2019b; Welch et al. 2021). In some cases, prolonged fasting may also alter the relative abundance and activity of specific microbial groups. However, because all animals underwent the same fasting protocol, the comparisons among treatments seem to remain valid, and the observed differences are interpreted as reflective of treatment effects rather than sampling bias.

The occurrence of low-grade liver abscesses and ruminitis was not significantly affected by treatment. According to Nagajara et al. (1997), monensin is effective in reducing lactic acid concentrations in the rumen, thereby lowering the risk of ruminal acidosis and associated lesions of the rumen wall. In the present study, all animals received monensin as part of their diet, which may explain the low incidence of ruminitis and liver abscesses across all treatment’s groups.

## Conclusion

The addition of 65 and 85 mg/kg DM to a high-concentrate finishing diet effectively reduced enteric CH_4_ emissions by 13.2 and 26.7%, respectively, without compromising animal health or performance. Despite the observed increase in DMI among Nellore bulls receiving 3-NOP, CH_4_ emissions per unit of DMI were significantly reduced, by 1.59 and 3.82 g of CH_4_/kg DMI for the 3-NOP65, 3-NOP85 treatments, respectively, compared to the control diet (15.56 and 13.33 vs. 17.15 g CH_4_/kg DMI). These reductions are likely associated with the decreased relative abundance of the phylum *Euryarchaeota*, which includes methanogenic archaea, in response to 3-NOP supplementation.

Metabolomic profiling revealed alterations in nutrient metabolism, suggesting enhanced nutrient utilization and metabolic efficiency in 3-NOP-supplemented animals. Although these metabolic shifts did not translate into measurable improvements in performance within the study period, it may be considered that they indicate potential for improved efficiency of feed utilization and productivity. These findings support the use of 3-NOP as a promising CH_4_ mitigation strategy in Brazilian beef production systems and highlight the need for further research to evaluate its long-term effects on animal performance and sustainability.

## Abbreviations

3-NOP: 3-nitrooxypropanol
ADG: average daily gain
BCAAs: branched-chain amino acids
CH_4_: methane
CP: crude protein
DM: dry matter
DMI: dry matter intake
MCR: methyl-coenzyme M reductase
NDF: neutral detergent fiber
OM: organic matter
PLS-DA: partial least squares discriminant analysis
SF_6_: sulfur hexafluoride
VFA: volatile fatty acids
VIP: variable importance in projection
